# Supplementary material for: Are Urologists Ready for Interpretation of Multiparametric MRI Findings? A Prospective Multicentric Evaluation
Source: Diagnostics (Basel). 2022 Nov 1;12(11):2656. doi: 10.3390/diagnostics12112656 (PMC9689928; doi:10.3390/diagnostics12112656)
Supplement: Supplementary file 1 [file diagnostics-12-02656-s001.zip › diagnostics-1949221-supplementary.pdf]

MULTICENTRIC STUDY PROSTATE MRI

AGE:

ROLE: RESIDENT (YEAR) UROLOGIST

INSTITUTION: UNIVERSITY HOSPITAL NON-UNIVERSITY HOSPITAL

EXPERIENCE IN PROSTATE BIOPSY? YES NO

EXPERIENCE IN FUSION PROSTATE BIOPSY? YES NO

NUMBER OF PROSTATE CASES DIAGNOSED YEARLY IN YOUR INSTITUTION:

NUMBER OF RADICAL PROSTATECTOMIES PERFORMED YEARLY IN YOUR INSTITUTION:

ARE YOU INVOLVED IN THE DIAGNOSIS AND MANAGEMENT OF PROSTATE CANCER PATIENTS? YES NO

DOES YOUR INSTITUTION HAVE A MULTIDISCIPLINARY TEAM FOR PROSTATE CANCER MANAGEMENT? YES NO
